# Designing a Policy Mechanism for Long-Duration Energy Storage: The British Experience

**DOI:** 10.1007/s40518-026-00287-y

**Published:** 2026-04-01

**Authors:** Elina Spyrou, Adam Suski, Richard Green

**Affiliations:** 1 Department of Electrical & Electronic Engineering, Imperial College London, London, United Kingdom; 2 Grantham Institute - Climate Change and the Environment, Imperial College London, London, United Kingdom; 3 Department of Economics and Public Policy, Imperial College London, London, United Kingdom

## Abstract

**Purpose of review:**

The UK parliament recently introduced a cap-and-floor mechanism for net revenues of Long-Duration Energy Storage in Great Britain (GB). The article summarizes learnings from the UK proceedings around four questions: (a) What drives the need for LDES? (b) What are the barriers to LDES deployment in GB? (c) Which options could mitigate these barriers? (d) What are the key design choices for the cap-and-floor mechanism?

**Recent findings:**

GB evidence indicates that (1) energy shifting over long timeframes and security-of-supply benefits drive LDES needs; (2) revenue uncertainty is the primary barrier; and (3) a cap-and-floor mechanism could mitigate investment risk.

**Summary:**

Evidence on LDES needs is extensive, whereas analysis of barriers, their impact on investment, and the effects of alternative policies and cap-and-floor designs remains limited. Further research could address this gap and contribute insights into the interplay of long-term contracts and short-term markets in hybrid electricity markets for deeply decarbonized power systems.

## Introduction

**Fig. 1:**
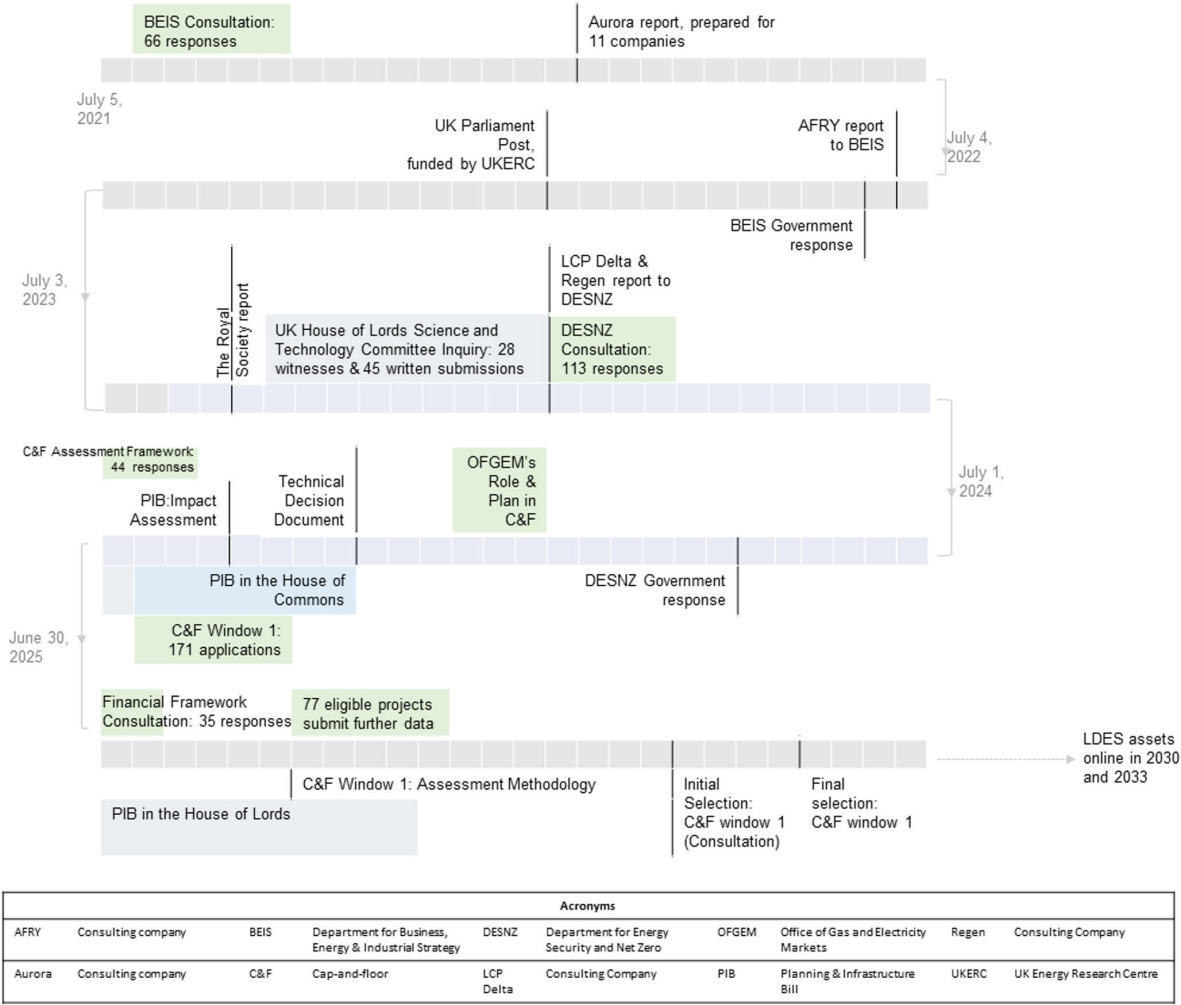
The timeline for development of the UK cap-and-floor mechanism for LDES. Note that the figure was finalized on 10 November 2025. Each row of grey boxes corresponds to a year, and each box is approximately 2 weeks long. The time progresses from left to right for the first year, then from right to left and so on. The ministry responsible for LDES was initially BEIS; DESNZ inherited its energy-related functions in February 2023

The UK has committed to reaching net zero by 2050 [1]. Achieving this goal requires a fundamental transformation of the power system. To integrate growing shares of electricity from variable renewable resources while serving rising demand, the power system in Great Britain (GB)^1^ will need greater flexibility, including storage solutions, which may need to have longer durations than typical utility-scale batteries [3]. Since 2021, various state agencies have gathered evidence and commissioned analyses to assess (a) whether there is a need for long-duration energy storage (LDES), defined in the UK as storage with a duration of more than 8 h^2^ [6]; and (b) whether barriers to LDES deployment may justify government intervention. This evidence-gathering process has been extensive, as it is evident in Fig. 1, which outlines key milestones, publications, and anticipated future developments. The process culminated in the introduction of a cap-and-floor mechanism for LDES in GB [6, 7].

In a net revenue cap-and-floor scheme, the floor guarantees a minimum net revenue that makes the project fundable, and the cap transfers excessive profits to consumers, reducing their energy bills. Net revenues are calculated by deducting market-related costs^3^ from gross revenues [8]. Applications to the LDES cap-and-floor scheme are accepted during prescribed application windows [9]. As the scheme is currently being applied for the first window, it offers a timely opportunity to provide early evidence and identify limitations and gaps that should be carefully considered in future research and policy proceedings in the UK and abroad.

This article focuses on four key questions that the evidence-gathering process aimed to answer: (a) What drives the need for LDES? (b) What are the barriers to LDES deployment in Great Britain? (c) What solutions could address barriers for LDES investments? (d) What are the key design choices for the cap-and-floor mechanism? The remainder of the article is structured around these four questions. We conclude the article with reflections on the process and recommendations for research and policy.

## What Drives the Need for Long Duration Energy Storage in Great Britain?

There is a clear economic case for short-duration battery storage providing intra-day arbitrage and ancillary services such as reserve and (very) fast response; more than 6.8 GW has been deployed in the UK to date [10]. The economic case for long-duration storage is less apparent, and the lack of recent investments prompted the government to gather evidence to understand whether “the electricity system requires and will benefit from LDES delivering […] services”.

Long-duration energy storage is a technology-agnostic concept encompassing assets with discharge durations exceeding a specified threshold [5, 11]. The choice of this threshold influences the relative importance of power-specific (in £/kW) versus energy-specific (in £/kWh) investment costs. For discharge durations between 8 and 100 h, compressed air energy storage, pumped hydro storage, and iron–air batteries are generally the most competitive technologies. For discharge durations exceeding 100 h, chemical storage (e.g., hydrogen storage) is expected to be the most cost-competitive option. Depending on the application, additional technological characteristics, such as round-trip efficiency and self-discharge rates, may influence the comparative advantage among options [4].

LDES deployment can play an important role in supporting multiple valuable applications [12]. In systems with large shares of variable renewable energy, it can be used to shift energy over long timeframes (between days, seasons, or even years [13]) and can reduce the need for peaking capacity [14]. Depending on its location, LDES can help reduce the impact of network constraints on system costs [15] and even reduce or defer transmission investment needs [16]. Depending on the control capabilities of LDES, it could also provide ancillary services for frequency and voltage control [17, 18].

LDES is not the only option for most (if not all) of these applications. Hence, approaches that holistically simulate the future energy system are necessary to quantify its benefits. Exploring the design space for LDES [19], finds that energy-specific investment costs and discharge efficiency greatly influence LDES’s peaking potential and its ability to shift energy over long timeframes [19]. also notes that projections for power-specific investment costs and roundtrip efficiencies are at a level that would *not* make LDES a competitive option for energy shifting over short timeframes (intraday). This finding holds for GB, where a least-cost capacity expansion study identified LDES as a competitive option for energy shifting over long timeframes (e.g., between seasons), but not for ancillary services and energy shifting over short timeframes [20]. Applications for which LDES represents the most cost-competitive option will drive its investment, but once deployed, LDES may also fulfil other roles. Accounting for the impact of LDES on system costs, the GB study projected 2050 LDES needs in the range of 2.5-3 GW^4^ (power capacity) and 12–17 TWh (storage volume^5^) [20].

Other GB-focused reports limited their scope to fewer applications. For instance [21], quantified needs in terms of the “energy available to be shifted” by LDES. Focusing on better utilization of renewable resources by minimizing renewable curtailments across 37 weather years [22], estimated needs for 60–100 TWh of storage volume, with a portion of this capacity used to shift energy across years. Results in [22] suggest that the system storage’s annual net discharge can be negative for up to three consecutive years. Energy shifting over such long timeframes appears possible in modelling studies that assume perfect foresight. However, in practice storage operators make decisions using imperfect, limited-horizon forecasts [23] and face pressure to meet annual or quarterly financial performance targets. Focusing on 2035 and 2050, [24] estimated how LDES could help reduce the need for additional peaking generation, manage network constraints, and shift energy. It also showed that LDES capital costs and the availability of other clean technologies, such as carbon capture and storage (CCS), critically impact the need for LDES. Both [20] and [24] indicated that non-hydrogen LDES, such as pumped hydro and compressed air energy storage, could be a bridge technology while the development of infrastructure, e.g., for hydrogen and carbon capture, is underway.

Beyond the applications already discussed, a report of the House of Lords Science and Technology Committee [25] highlighted interest in LDES for its role in mitigating energy supply shocks triggered by geopolitical events or extreme weather. In that report, LDES is positioned as the low-carbon counterpart to natural gas storage in net-zero energy systems. Under this framing, one might expect LDES requirements to mirror historical levels of gas storage in the UK and abroad. For reference, France, Germany, and Italy can store 100–250 TWh of natural gas each (~ 30% of their annual consumption) [26], although the UK, with its domestic production, only has around 30 TWh of storage capacity (~ 5% of the 2023 UK demand) [27]. Therefore, projections of LDES needs in the range of 10–100 TWh appear reasonable. However, some drivers for natural gas storage, such as reliance on imported fuel, are not relevant in systems where domestic renewable resources generate most electricity.

In summary, multiple analyses in GB showed that energy shifting over long timeframes, along with benefits for security of supply in an energy system with close to zero greenhouse gas emissions, drives the need for LDES. Consistent with the broader literature [13, 19], GB studies also highlighted three critical interactions: (1) the scale of LDES needs is highly sensitive to assumptions about capital costs, (2) the need for LDES increases when the deployment of CCS is more limited, (3) the assessed need for LDES is sensitive to the variability among the weather years considered. Whereas the reports offered several insights into the magnitude of the need, they lacked the financial predictions for LDES that could have provided a starting point for a constructive discussion on the barriers to LDES investments and the design of state-backed mechanisms.

## What are the Barriers to LDES Deployment in Great Britain?

The evidence on the drivers for LDES needs indicates a broad consensus that *additional* LDES capacity would benefit the future GB energy system. Yet, in practice, private investment in LDES remains extremely limited. The last major LDES asset built in GB was the Dinorwic pumped hydro storage scheme, completed in 1984, with only a handful of small projects recently supported through the Longer Duration Energy Storage (LoDES) Demonstration Programme [28]. This raises the central policy question: are there barriers that make government intervention necessary?

To answer this question, the government engaged with academics, developers, investors, and industry bodies. These engagements [29, 30] highlighted a wide range of barriers: high capital costs, partially caused by the lack of economies of scale in LDES development; long lead times; lack of a commercial track record; revenue uncertainty; weak market signals for multi-day arbitrage (i.e., low price volatility); insufficient consideration of carbon prices in flexibility markets; long grid connection times; and distortions created by government support for other flexible technologies or through cross-subsidies in network charges.

Among these barriers, the government prioritized ‘revenue uncertainty’ as the most significant one to address through policies [6]. This was a sensible choice for a variety of reasons. Some of the other barriers, such as long connection times, affect all new entrants and call for system-wide reforms rather than LDES-specific policy. Others are unintended consequences of legacy market design. For example, LDES lead times were longer than lead times in the capacity market. For this type of issue, it is preferable to revise the design of that existing market, e.g., allowing for an extended delivery year for LDES participating in the capacity market, rather than introducing an LDES-specific policy. The barrier of limited track record applies primarily to emerging LDES technologies, and it is likely irrelevant for mature technologies such as pumped hydro storage.

Whereas revenue uncertainty is a major barrier, we find its framing quite generic. The original proceedings [29] note under revenue uncertainty that ‘projects with high up-front capital costs and long lead times generally require more *visible* long-term cash flows to secure finance and investment.’ and later proceedings claim that “Although LDES assets are *likely* to be *independently profitable* once developed, the initial risk, primarily from high upfront capital costs, is too high for private investors to bear without Government support.” [31].

The *visibility* over (or likelihood of) long-term cash flows could be obscured by multiple factors such as policy uncertainty (e.g., regarding future electricity market arrangements [32]) and technological uncertainty (e.g., regarding evolution of competing technologies such as Inverter-Based Resources for ancillary services and short-duration batteries for energy arbitrage and frequency regulation). These uncertainties can create ambiguity regarding the nature and significance of different revenue streams. The record shows divergent views on LDES revenues related to ancillary services [20, 21] and concerns about insufficient price volatility to ensure profitability through energy shifting over long timeframes.

General lack of liquidity in long-term electricity markets [33] could also explain a project’s inability to secure financing despite projections that show it as *independently profitable*. The more volatile the returns of an *independently profitable* project the higher its cost of capital is and the more severely the project is affected by missing risk markets. To de-risk the investment, the government (on behalf of consumers) can intervene and reduce the revenue volatility seen by the investor. There are at least two possible reasons for such an intervention. First, the government is less risk-averse than private investors. Second, risk pooling could result in reduced volatility of both consumer costs and LDES revenues, but retailers hesitate to sign long-term contracts with LDES. Previous analysis suggests that retailers might be reluctant to commit to seemingly ‘win-win’ long-term advance purchase arrangements because they risk being out-of-the-money should wholesale prices fall, having to absorb the loss, or risk losing customers to rivals buying energy in the spot market [34–36].

In summary, revenue uncertainty is perceived as the major barrier to LDES investment in GB. However, the barrier description is so generic that it could point to several different root causes, ranging from policy and technological uncertainty to missing money for arbitrage over long timeframes and missing markets for long-term contracts and risk management. There is a clear need for future research on simulation of barriers to understand the extent to which they affect LDES investments. Recent literature that simulates the dispatch of LDES assets and the effects of missing risk markets on investments [37–40] could be leveraged and further extended.

## What Solutions Could Address Barriers to LDES Investments?

To address the barriers to LDES investments, the Department of Energy Security and Net Zero initially considered fourteen options [41]. The Department evaluated them using four criteria: (a) incentives for market flexibility; (b) revenue certainty for LDES; (c) government costs; and (d) timing to support entry by 2030. In parallel, a study [21], produced by Aurora for a group of companies in the public and private sector, assessed a subset of options using similar criteria: (a) incentives for efficient LDES dispatch; (b) investor confidence; (c) prevention of market distortions; and (d) accelerated deployment.

We present ten of these options in Table I^6^ and summarize the arguments presented in [41] and [21] using three general criteria for policy interventions—**effectiveness**,** responsiveness**,** and coherence** [42].

- **Effectiveness** measures the extent to which regulation achieves its objective, considering costs, loopholes, and creative adaptation. This aligns with criteria focused on government costs, investor confidence, and revenue certainty.
- **Responsiveness** refers to a regulation’s ability to maintain desirable social practices and ensure welfare outcomes. Both reviews captured this through their focus on incentives for flexibility, efficient dispatch, and avoidance of market distortions.
- **Coherence** concerns consistency, predictability, and adherence to regulatory principles. Interestingly, “strategic policy alignment” was added as a criterion when the final bill was drafted, and it refers to coherence of policies by the Department of Energy Security and Net Zero and alignment with decarbonization and energy security goals. Considering the decarbonization goals for 2030, timing and accelerated deployment are discussed under this criterion.

The options fell into three categories: fiscal policies, revisions of electricity market arrangements, and policy mechanisms. Fiscal policies such as investment grants were quickly discarded, partly because budget revisions would delay legislative approval, and partly because they were deemed insufficient to address revenue uncertainty. This highlights the importance of clearly articulating the barrier. If revenue uncertainty is understood as the absence of price signals for multi-day arbitrage, fiscal policies are ineffective. If, instead, the issue is a missing risk market, government-backed loans or guarantees could appear more suitable.

Most proposed market reforms were also set aside, largely because rules and products could not be designed and implemented quickly enough to influence development decisions for projects expected to be operational in 2030. To date, the literature offers robust evidence only for capacity remuneration mechanisms such as strategic reserves [43] and capacity markets [44]. Options also included concepts of short-term products, such as a sustained response balancing service. These concepts have not been discussed in the literature, which may indicate either limited merit in pursuing them or, alternatively, an opportunity for future research. At the same time, the solution set did not include enhancements such as markets with an extended horizon that the literature has found effective for LDES dispatch [45].

Interestingly, the arguments presented reveal two possible meanings of the ‘incentives for market flexibility’ criterion. The first interpretation captures any distortionary effects of a policy on the market incentives for dispatch. For example, a storage asset with a Contract-for-Differences for energy discharged is incentivized to cycle a lot and ‘generate’ as many MWh as possible, which is not necessarily the optimal way of dispatching the LDES for the system. The second interpretation implies the presence of a missing money problem for energy shifting over long timeframes, which was not identified by simulations of the future GB system [20]. This missing money problem may be driven by market design features (e.g., related to operating reserves) that can suppress price volatility [46] or by imperfect price forecasting capabilities [47]. Hence, if simulations omit those market design features or follow unrealistic assumptions about foresight, the missing money problem will likely appear when new LDES assets connect to the grid, making it a priority to proactively evaluate market and dispatch reforms.

Turning to policy instruments, the literature provides some guidance on their relative effectiveness for supporting LDES investments [47, 48]. In GB, decision-makers gravitated toward policy mechanisms already applied for other clean technologies, with their costs recovered through energy bills. The use of familiar mechanisms supports coherence and timely delivery, but it risks poor fit, given the unique characteristics of LDES. Poor fit explains why Contract-for-Differences for energy output (i.e., electricity discharged)^7^ and regulated asset base^8^ models were quickly dismissed. These give an asset a (largely) fixed revenue stream despite fluctuating market prices, but LDES must be able to pass on the similarly variable cost of charging.

Ultimately, the cap-and-floor option, along with minor revisions in the capacity market to limit barriers to entry, was selected. Table 1 indicates that the net revenue cap-and-floor mechanism was chosen as the only option that could be implemented promptly while boosting investor confidence by addressing revenue uncertainty. The government chose pace over perfection by not analysing the impact of the scheme in detail and trusted that a carefully designed cap-and-floor scheme would avoid any negative effects on market efficiency and outcomes for consumers.

**Table 1:** Summary of policy options and synthesis of arguments in favour or against them, summarized across three criteria. Symbols (+) and (-) indicate strengths and weaknesses, respectively. In cases where an argument is ambiguous (i.e., it could be perceived as a strength or weakness), we have used bold font to indicate which part of the argument is the basis for our assessment

	Effectiveness	Responsiveness	Coherence
Fiscal policies			
Capital grant	(+) Reduces amount of private capital (-) Does not address revenue uncertainty (-) Cost transfer to taxpayers and immediate impact on government budget	(-) Does not strengthen operational/market signals for flexibility over long timeframes (+) Does not distort effects of existing operational/market signals	(-) Unlikely to be implemented on time for achieving decarbonisation goals
Government investment	(+) Reduces amount of private capital (-) Does not address revenue uncertainty (-) Risk transfer to taxpayers and immediate impact on government budget		
Government loan	(+) May reduce cost of capital (-) Does not address revenue uncertainty (-) Risk transfer to taxpayers and immediate impact on government budget		
Government guarantee	(+) May reduce cost of capital (-) Does not address revenue uncertainty (-) Risk transfer to taxpayers and potential long-term impact on government budget		(-) Less likely to be implemented on time for achieving decarbonisation goals
Electricity market arrangements			
Capacity market: extended delivery year	(+) Removes barriers to entry for technologies with long build times (-) Additional revenue stream of **uncertain magnitude**	(-) Does not strengthen operational/market signals for flexibility over long timeframes (+) Does not distort effects of existing operational/market signals	(+) Enhancement within current arrangements (+) Can be implemented on time for achieving decarbonisation goals
Sustained Response Balancing Service (concept)	(-) Additional revenue stream; **unclear impact** on revenue certainty	(+) It conceptually addresses issues with weak incentives for flexibility over long timeframes	(-) Unclear how this service fits within the service portfolio of the system operator (-) It would take a long time to design and implement, making the timely achievement of decarbonisation goals difficult
Strategic reserve	(+) Revenue certainty (-) Likely high cost borne by electricity consumers	(-) It can negatively affect market efficiency by under-utilizing assets classified as strategic reserves	(-) Significant change to market arrangements (-) Highly unlikely that this could be implemented on time for achieving decarbonisation goals
Policy mechanisms			
Regulated Asset Base (RAB)	(+) Guaranteed return on investment, which likely reduces the cost of capital (-) Cost and risk (potentially excessive) borne by electricity consumers	(-) Distorts incentives of asset to respond to market and operational signals (-) Does not strengthen operational/market signals for flexibility over long timeframes	(-) Unlikely that the legislation can be developed on time for achieving decarbonisation goals
Incentive payments (concept): pay storage to charge (and/or dispatch) or guarantee premium over charging costs	(-) Additional revenue stream; **does not provide** revenue certainty (-) Ongoing cost and risk for electricity consumers or taxpayers	(+) It conceptually addresses issues with weak incentives for flexibility over long timeframes	(-) New policy revenue stream **with additional regulatory and administrative burden** (-) Unclear if it could be implemented on time for achieving decarbonisation goals
Net revenue cap-and-floor	(+) Certainty about minimum net revenue (-) Potential for balanced sharing of risk between consumers and LDES owners/investors. However, **consumers may incur significant costs to top up net revenues depending on market conditions**	(-) Limited **distortions** on market participation of LDES (-) Does not strengthen operational/market signals for flexibility over long timeframes	(+) Experience with the interconnector C&F scheme (+) Likely timely implementation for achieving decarbonisation goals

## What are the Key Design Choices for the Cap-and-Floor Mechanism?

A net revenue cap-and-floor scheme reduces uncertainty about the return on LDES investment throughout the scheme’s duration by guaranteeing a minimum net revenue to secure financing and transferring excessive profits to consumers. Table 2 summarizes the main design choices of the LDES cap-and-floor mechanism, and a comprehensive description of the mechanism is provided in [8]. Most design choices regarding the cap and floor levels [8], the assessment methodology [50], and the contract terms have been based on qualitative reasoning and stakeholder input, with limited use of stochastic modelling for illustrative cases [51]. OFGEM, the GB energy regulator^9^, has been appointed to administer the LDES cap-and-floor scheme, drawing on its prior experience with a similar framework for interconnectors [52].

**Table 2:** Key design choices for the cap-and-floor scheme^10^

**Design choice**	**Should**:
Cap	• Be high enough to incentivize efficient market integration • Be low enough to protect consumers
Floor	• Be high enough to secure finance and investment • Be low enough to incentivize efficient market integration
Procurement process	• Consider criteria regarding costs and benefits that are mutually exclusive and comprehensively exhaustive • Evaluate each criterion based a transparent and reproducible method • Use a transparent and clear method to weigh criteria • Use a mechanism to choose winning applications that encourages truthful bids • Target technologies that face the barriers the scheme aims to overcome
Contract terms	• Address gaming opportunities and minimize risk of market distortions • Incentivize timely construction • Include stipulations for edge cases such as projects with longer lifetimes than scheme duration

For a full list of design choices, please refer to [7, 9]

The experience with interconnectors is indeed valuable for determining the cap and floor levels. On the one hand, the cap needs to be low enough to protect consumers. On the other hand, too low a cap might discourage efficient market integration. In practice, a soft cap allowing the sharing of revenues between consumers and investors is commonly used to maintain strong operational incentives for assets when net revenues exceed the cap. In this case, the level of the sharing parameter for excess net revenues was increased from 10% to 30%, based on inputs from stakeholders. Similarly, the floor needs to be high enough to secure finance and investment but low enough to encourage effective operation for the supported asset. For interconnectors, the level of the floor depends on the availability of the asset. However, for LDES, determining availability or minimum performance requirements is not straightforward. A hard floor has been chosen and further work is planned to define project-specific availability metrics and targets [9].

Process-wise, projects can apply for support during specific application windows. The LDES scheme aims to select projects to meet a target long-duration energy storage (LDES) capacity (in MW and potentially MWh)^10^ under each window. While the first application window is already underway, its target capacity remains undisclosed, as do the timelines and capacity targets for subsequent windows. Providing this information in advance would offer investors greater clarity and enable more informed decision-making. The regulator should therefore consider publishing such details well ahead of future application rounds.

Like the scheme for interconnectors, the assessment process for LDES aims to holistically capture the costs and benefits of supported assets. However, the number of LDES applications, 171 in the first window, is much higher than that of interconnectors, requiring a practical approach [54]. The current process includes three steps: eligibility screening, selection, and post-construction contract evaluation. Defining eligibility criteria presents a delicate balance. Overly strict requirements risk too few viable applications, which could raise the cost of the mechanism and reduce system efficiency. Conversely, criteria that are too relaxed may attract applications that are not genuinely affected by the identified barriers, resulting, at best, in increased work to disqualify applications or, at worst, reducing the effectiveness of the scheme by supporting the wrong projects.

The selection step is at least as important to the success of the scheme as the eligibility screening. The scheme follows principles of hybrid market design, creating competition for the cap-and-floor support and relying on short-term liberalized markets for effective operations [55]. The selection step could be interpreted as a multi-attribute auction, in line with the interpretation of electric utility procurement processes as multi-attribute auctions by Laffont and Tirole [56]. Applicants submit data forms with information on their projects, which can be viewed as multi-dimensional bids. The evaluation of the attributes is performed through a combination of three assessments (Economic, Strategic, and Financial). The economic assessment includes sixteen criteria, nine of which are in monetary terms, six qualitative, and one quantitative. The strategic assessment evaluates how a supported project contributes to six attributes of the portfolio of projects supported by the scheme: technological and locational diversity, risk of cost overruns, deliverability, interdependency between projects, and flexibility across scenarios. Finally, the financial assessment ensures that only financially viable projects are supported. Initially, the regulator considered that it could encourage competition by allowing applicants to submit their target rate of return, proposed residual value of the project, regime duration, interest during construction, and decommissioning cost. However, after strong opposition from stakeholders and consideration of practical complications, the regulator decided to encourage competition for two parameters (regime duration and residual value) and set the remaining three parameters administratively [8].

Assessing the criteria in terms of completeness, redundancy, operationality, mutual independence of preferences, double-counting, size, and temporal evolution [57], we offer some observations that might help further revisions and developments. First, the list of criteria is long, indicating an aspiration to be comprehensive and thorough, but omitting evidence collected so far. For instance, given findings by [20], the list could be shortened by excluding criteria related to real-time flexibility and system operability that were found to be inconsequential for LDES. The lack of a common, replicable methodology makes several criteria operationally challenging. Instead of relying on applicant-proposed methodologies and qualitative estimates, a standard approach would promote transparency and reduce application burden. Stakeholders also raised similar concerns about the strategic assessment [58]. To make the strategic assessment more systematic in future windows, the regulator could explore tools such as multi-stage capacity expansion planning [59]. Scrutinizing criteria to consider their inter-dependency would also clear ambiguities about double-counting. For instance, avoided renewable curtailment, costs for policies supporting renewable energy, and unpriced carbon externality costs are listed as three separate criteria, but their performance is likely correlated.

Joskow underscores the importance of good auction design [55]. At a high level, the regulator aims to select the most beneficial projects that add up to the target capacity [50] or a quantity exceeding the target to compensate for any undeliverable projects. The economic assessment will rank projects based on their benefit-to-cost ratio, and the financial assessment will be used to break any ties between projects. However, the influence of the strategic assessment on the ranking and the eventual allocation is ambiguous. The regulator has retained the right to revise the process at any time as long as it is transparent and gives stakeholders the opportunity to represent themselves. Consequently, the principal critique that can be raised now is that the process lacks clarity, specificity, and predictability.

Whereas the assessment for the first window is underway, key license terms aiming to prevent market distortions, close off gaming opportunities, and mitigate project risks, such as credit exposure, have not been finalized. For instance, it is unclear whether LDES will participate as a price-taker in the capacity market [60]. The assessment methodology [50] assumes that LDES does not set the price in the capacity market. In a future where LDES is expected to have a major role in system security, this restriction could lead to lower capacity market-clearing prices relative to a counterfactual in which LDES participates and sets the price, thereby increasing the likelihood of reduced returns (and potentially increasing the reliance on floor payments) for LDES. This ambiguity about license terms affects not only LDES, but also other market participants who cannot project with confidence revenue streams for which they might compete with LDES assets supported by the cap-and-floor scheme.

Upon construction completion, the cap and floor will be finalised based on the actual construction costs incurred. At the application stage, projects submit three cost estimates (low, central, high). The central estimates inform preliminary cap and floor levels. Any cost overruns must be robustly evidenced, and overruns exceeding the highest estimates are subject to additional regulatory scrutiny.

The review underscores the dimensionality and complexity of policy design. Considering the accelerated timeline, the efforts of the energy regulator and the National Energy System Operator are commendable. Nevertheless, the proposed process is far from perfect, and it is essential to further specify it and apply it with caution, potentially for a small volume of LDES capacity at first.

## Conclusions

The process that has culminated in the cap-and-floor mechanism for long-duration energy storage in Great Britain carries a few takeaways for future endeavours. In Great Britain, the government stepped in to de-risk the investment through a cap-and-floor mechanism based on evidence that: **(1)** more long-duration energy storage is needed; **(2)** deployment faces persistent barriers; and **(3)** among the policy options considered, the cap-and-floor mechanism could be implemented in a timely manner and could strike a good balance regarding the risk shared between consumers and investors, without directly distorting electricity markets.

Our review reveals a disproportionately larger evidence base on the need for LDES and the impact of different LDES capacities on the British power system. In contrast, the evidence base lacked quantitative analyses on the barriers, their relative impact on investment and consumers, and the effects of alternative policy mechanisms. Depending on the framing of the barrier, the relative merit of alternative regulatory decisions changes. For instance, if a lack of risk markets hinders risk management for LDES, fiscal instruments were likely dismissed too quickly, in favour of policies that pass their costs to consumers through energy bills, with potentially regressive outcomes. Considering that some risk is borne by consumers, consumer participation or representation in consultations becomes critical. Whereas market reforms alone would not address all barriers LDES face, it is still worth reflecting on why market solutions were deemed as impractical, and whether they should be pursued to address operational challenges that will likely appear once LDES connect to the system.

Over the first six months of 2026, the GB energy regulator will assess seventy-seven eligible projects (see Fig. 1 and [61]), gaining practical experience on how well its approach promotes the fundamental objectives of energy policy. A consultation on an initial selection of projects is planned for spring 2026, with the final list of projects expected to be announced in summer 2026. A consultation on license conditions is also expected in summer 2026, as contractual details are expected to be final by June 2026 [9]. Going forward, the ambitious timeline for decarbonization in Great Britain will prompt policymakers to face similar dilemmas between intervening to accelerate delivery and reforming market arrangements to achieve market-driven transformation. It will be interesting to see if the accelerated decarbonization goals will push market arrangements towards hybrid markets with a more pronounced role for long-term tools that encourage competition for the market. The review of the policy proceedings highlights an opportunity for rigorous academic work to contribute insights into the role of resilience as a driver for LDES, potential barriers and business models for clean energy technologies, and performance of novel market designs and policy mechanisms for net-zero systems. Finally, with 2050 fast approaching, this may be the right moment to adopt a pragmatic approach and design adaptive regulations that evolve with innovations co-created by industry and academia.

## Data Availability

No datasets were generated or analysed during the current study.

## Footnotes

1 Great Britain (England, Scotland and Wales) has a separate electricity system from Northern Ireland. UK parliamentary bills on energy policy mainly cover Great Britain, as “Energy policy is largely devolved to the Northern Ireland Executive (NIE)” [2].

2 The threshold of eight hours is not universally employed, and more generic definitions classify a technology as LDES based on the level of its energy-specific investment costs [4, 5].

3 For LDES, cycling costs are market-related and will likely be deducted from gross revenues subject to regulatory approval [8].

4 Note that the needs correspond to the minimum capacity identified across scenarios and they do not cover power capacity for charging/discharging hydrogen storage (e.g., electrolysers).

5 We use the term volume to refer to the energy stored. For example, in [20], “The hydrogen volumes of storage here [11.4TWh to 17.2TWh] are sufficient to allow for around 6-9TWh of production from hydrogen Combined Cycle Gas Turbines (CCGTs) .”

6 Table 1 does not include three policy mechanisms that have been designed for technologies drastically different from long duration storage and changes discussed under the Review of Electricity Market Arrangements (REMA).

7 A CfD that fixed the output price received by LDES but required it to pay the market price for energy used in charging would expose it to the risk of bankruptcy if that price rose. There are multiple forms of Contract-for-Differences (CfD), including newly proposed CfD for storage [48]. The UK has used CfDs for actual energy output for renewable generation and initially considered it as an option for storage, where the energy output would be the amount of electricity discharged. It is worth noting that the poor fit was identified for a particular form of CfD and it is not generalizable to all forms of CfDs [49].

8 A Regulated Asset Base scheme, which sets revenues so that an efficient company can achieve a suitable return on its assets, requires that the business has predictable costs, which is not the case for LDES.

9 Northern Ireland Authority for Utility Regulation is the energy regulator in Northern Ireland.

10 Point 6.27 in [53] states “Ofgem will set a target capacity range in MW for Window 1, based on updated advice from NESO (National Energy System Operator). We may also separately define a minimum MWh threshold depending on the nature of the advice. “.
